# Emerging experimental and bioinformatic approaches in RNA interference‐based pest control research

**DOI:** 10.1111/imb.70040

**Published:** 2026-04-07

**Authors:** Doga Cedden

**Affiliations:** ^1^ Department of Evolutionary Developmental Genetics, Göttingen Center for Molecular Biosciences University of Göttingen, Johann‐Friedrich‐Blumenbach Institute Göttingen Germany; ^2^ Branch Bioresources Fraunhofer Institute for Molecular Biology and Applied Ecology IME Giessen Germany

**Keywords:** design, dsRNA, efficacy, methodology, off‐target, RNAi, tool

## Abstract

RNA interference (RNAi) has emerged as a promising strategy for species‐specific and environmentally friendly pest control, offering an alternative to conventional chemical insecticides that are increasingly constrained by resistance development and ecological concerns. RNAi‐based approaches involve oral delivery of double‐stranded RNA (dsRNA), which is processed into RNA‐induced silencing complex (RISC)‐bound small interfering RNA (siRNA) to silence essential genes of pests. This review synthesizes recent advances in experimental and bioinformatic methodologies that are facilitating and enhancing RNAi research in insect pest management. Particular emphasis is placed on molecular validation techniques that move beyond phenotype‐based bioassays, including RISC‐bound small RNA sequencing to resolve dsRNA processing and guide strand selection, RNA degradomics to map siRNA‐mediated transcript cleavage events and transcriptomic and proteomic profiling to characterize genome‐wide responses and compensatory effects. In parallel, dsRNA visualization methods provide mechanistic insight into uptake, intracellular trafficking and degradation dynamics, clarifying barriers that distinguish responsive from recalcitrant species. Complementing these experimental developments, emerging computational platforms enable insect‐optimized target selection, dsRNA design and environmentally informed off‐target prediction. Together, these innovations support a transition toward more predictive and mechanistically grounded RNAi‐based pest control applications. The integration of high‐resolution molecular tools with specialized bioinformatic pipelines is expected to enhance efficacy, safety and reproducibility, advancing RNAi‐based pest control toward practical and scalable agricultural deployment.

## Graphical abstract

- Double‐stranded RNA (dsRNA) visualization, RNA‐induced silencing complex (RISC)‐bound sRNA‐seq and RNA degradomics allow investigating dsRNA uptake and processing, guide small interfering RNA selection and RISC‐mediated target mRNA cleavages.
- Transcriptomic and proteomic profiles of genome‐wide responses to dsRNA, including off‐target and compensatory effects.
- Emerging web platforms and tools enable insect‐optimized target selection, dsRNA design and safety evaluation.

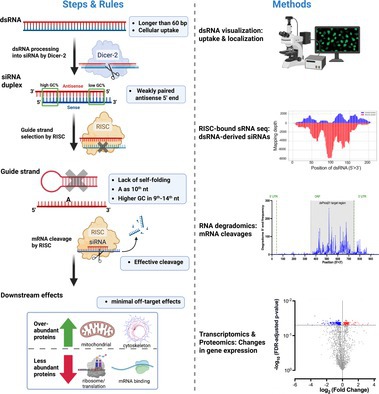

## INTRODUCTION

The growing global population continues to intensify pressure on agricultural production, demanding pest management strategies that are both effective and environmentally sustainable (Deguine et al., 2021; Deutsch et al., 2018; Gu et al., 2021). Conventional chemical insecticides have long served as the main pillar of pest control, largely due to their high efficacy and broad‐spectrum activity against insect pests (Sparks & Nauen, 2015). However, their extensive use has driven widespread resistance in pest populations and raised substantial environmental concerns, including chemical residues and detrimental effects on pollinators (Lundin, 2021; Scott & Bilsborrow, 2019; Sparks et al., 2020). These issues have motivated the search for eco‐friendly alternatives with high‐species specificity and reduced ecological impact. RNA interference (RNAi)‐based pest control has emerged as a promising alternative, with recent commercialization successes and ongoing research targeting many important pests and pathogens (Huvenne & Smagghe, 2010; Reinders et al., 2023; Rodrigues et al., 2021).

**FIGURE 1:**
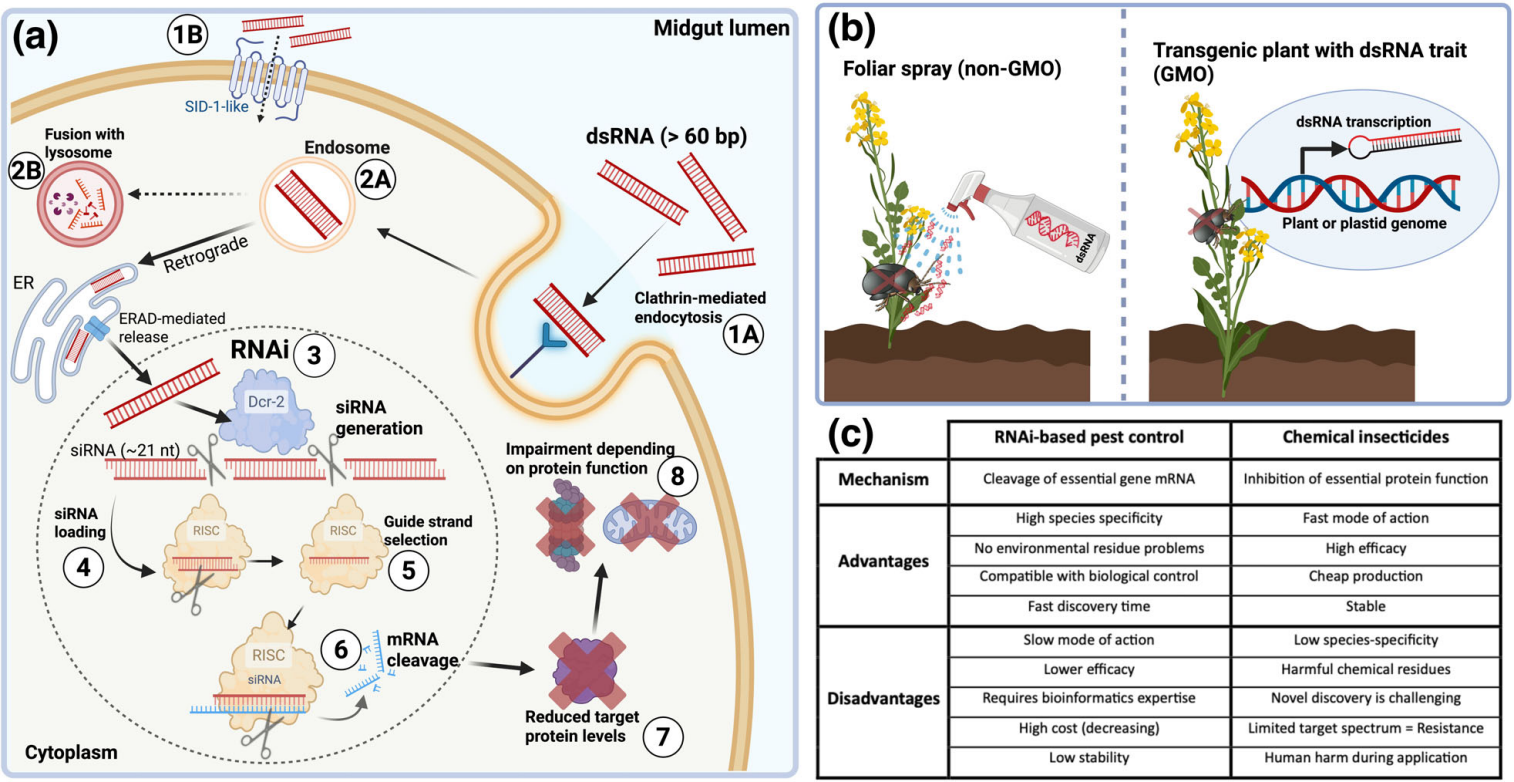
Overview of RNAi‐based pest control. (a) Molecular mechanism of RNAi in insects. (1A) Double‐stranded RNA (dsRNA) longer than 60 base pairs (bp) enters cells mainly through clathrin‐mediated endocytosis. (1B) Alternative, though less supported, entry pathway involves SID‐1‐like membrane proteins. (2A) Endocytosed dsRNA must be released into the cytosol to be processed by the RNAi machinery. Recent evidence in *Leptinotarsa decemlineata* suggests that endocytosed dsRNA is first transported to the endoplasmic reticulum (ER) via the retrograde pathway and subsequently released into the cytosol, facilitated by the ER‐associated protein degradation (ERAD) pathway (Koo & Palli, 2024, 2025). (2B) If the dsRNA remains in the endosome, it is degraded after fusion with lysosomes, preventing RNAi induction. (3) In the cytosol, the endonuclease Dicer‐2 (Dcr‐2) processes dsRNA into ~21 nucleotides small interfering RNA (siRNA) duplexes. (4) siRNA duplex is loaded into the RNA‐induced silencing complex (RISC). (5) During RISC maturation, one strand (guide strand) is retained, while the passenger strand is discarded. (6) Mature RISC uses the guide strand to recognize and cleave complementary messenger RNAs (mRNAs) or other RNAs, such as viral genomes, resulting in sequence‐specific degradation. (7) Amount of protein encoded by the target mRNA is reduced. (8) Reduced protein levels impair cellular functions, which can lead to lethality if the target gene has essential functions. (b) RNAi‐based pest control strategies. RNAi‐based pest control relies on oral uptake of dsRNA targeting essential genes. Delivery methods include foliar application of dsRNA formulations or cultivation of transgenic plants producing dsRNA. Foliar sprays are particularly relevant in regions where genetically modified crops are restricted (e.g., Europe). (c) Comparison with chemical insecticides. RNAi‐based pest control is highly species‐specific and environmentally safer but has slower mode of action and lower efficacy. Conventional chemical insecticides act faster but cause environmental harm, including effects on pollinators and natural enemies. Created with BioRender.com. bp, base pairs; Dcr‐2, Dicer‐2; dsRNA, double‐stranded RNA; mRNA, messenger RNA; nt, nucleotides; RISC, RNA‐induced silencing complex; RNAi, RNA interference; siRNA, small interfering RNA.

The RNAi pathway is initiated by the recognition of double‐stranded RNA (dsRNA) by Dicer‐2, which is an endoribonuclease belonging to the RNase III family (Figure 1a) (Chen & Hur, 2022; Kim et al., 2006; Yamaguchi et al., 2022). Dicer‐2 cleaves dsRNA into a pool of ~21 nucleotide‐long small interfering RNA (siRNA) duplexes (sense/antisense) with characteristic two‐nucleotide overhangs at their 3′ ends (Su et al., 2022). Typically, a single dominant siRNA length is observed for each insect species, with 21 nucleotides being the most common (Cedden et al., 2024; Su et al., 2022). Following the generation of the siRNA duplexes, both strands are initially loaded into Argonaute‐2, forming the RNA‐induced silencing complex (RISC) (Czech et al., 2009; Iwakawa & Tomari, 2021; Preall & Sontheimer, 2005). RISC discards one of the strands of the siRNA duplex, referred to as the passenger strand, and retains the other as the guide strand. Next, RISC recognizes and cleaves complementary RNA molecules at the phosphodiester bond opposite the 10th and 11th nucleotides of the guide strand (Haley & Zamore, 2004; Iwakawa & Tomari, 2021).

RNAi‐based pest control involves the delivery of dsRNA complementary to an essential gene of the target pest (Baum et al., 2007; Cedden & Bucher, 2025). Two main strategies have been explored for dsRNA delivery in the field (Figure 1b): cultivation of transgenic plants engineered to express dsRNA (e.g., SmartStax® PRO maize against the Western corn rootworm *Diabrotica virgifera virgifera*) and exogenous applications of sprayable dsRNA formulations (e.g., Ledprona against the Colorado potato beetle, *Leptinotarsa decemlineata*) (Joga et al., 2016; Reinders et al., 2023; Rodrigues et al., 2021). The uptake of the dsRNA into pest cells results in the induction of the RNAi pathway and consequently the silencing of the target essential gene. The silencing, in turn, depletes the targeted protein levels, causing cellular impairments and severe insecticidal effects, including feeding inhibition and death (Cedden et al., 2024; Cedden, Güney, Debaisieux, 2025; Rodrigues et al., 2021). RNAi‐based pest control is more environmentally friendly and safer than chemical insecticides, because (a) sequence‐specificity of RNAi often results in higher species‐specificity (Whyard et al., 2009), (b) dsRNA molecules are naturally present in foods we consume and in virtually all organisms (Borges & Martienssen, 2015; Chen & Hur, 2022), (c) being a natural molecule, dsRNA decays rapidly and does not leave harmful residues (Bachman et al., 2020) (Figure 1c).

Current research in the field primarily focuses on establishing important pest species as targets of RNAi‐based control, improving RNAi responses in recalcitrant insects and conducting basic research on how exogenous dsRNA is taken up and processed in insect pests (Cedden et al., 2024; Cedden & Güney, 2026; Mehlhorn et al., 2021; Shi et al., 2026; Wei et al., 2022). To these ends, recent studies have begun to incorporate and develop elaborate experimental and bioinformatics tools. Emerging bioinformatic pipelines increasingly automate target selection and dsRNA design, thereby facilitating RNAi research in new pest species. In parallel, recent developments in experimental methods provide unprecedented insights into the mode of action of RNAi‐based pest control at the molecular level. Together, these advances are contributing to the transformation of RNAi from a promising concept into a more feasible and predictable pest management strategy. This review summarizes emerging experimental and bioinformatics approaches that have been applied to RNAi research in insect pest control, with particular emphasis on strategies that clarify the molecular mechanisms of RNAi and improve dsRNA design.

## EXPERIMENTAL APPROACHES

**FIGURE 2:**
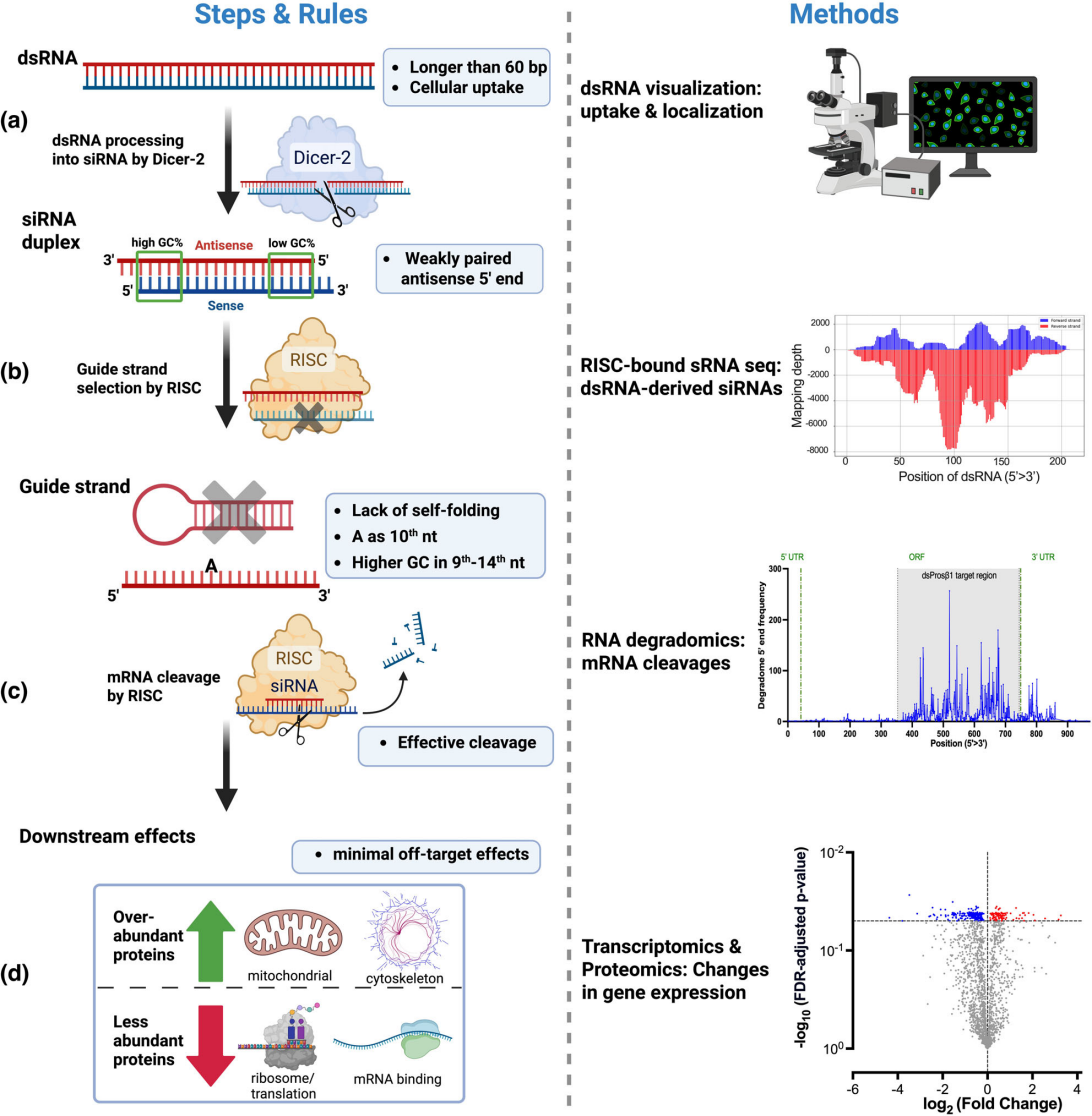
Steps in the RNAi pathway, design rules and analytical methods. (a) Insect cells uptake long dsRNA, which can be assessed through tagged dsRNA visualization. (b) dsRNA is processed by Dicer‐2 into siRNA duplexes in the cytoplasm. RISC loading drives guide strand selection, a critical determinant of silencing because only antisense siRNAs are complementary to the target transcript. The 5′ end of the antisense strand should be weaker than the 3′ end for preferential loading and high RNAi efficacy. dsRNA processing and guide strand selection dynamics can be analyzed through RISC‐bound small RNA‐seq. Exemplary data show how a dsRNA is processed into RISC‐bound sense (blue) and antisense (red) siRNAs (Cedden, Güney, Rostás, & Bucher, 2025). (c) Guide siRNA strand directs the RISC to cleave complementary RNAs. Exemplary data show how a dsRNA‐complementary transcript is cleaved at multiple sites within the target region (grey) (Cedden, Güney, Rostás, & Scholten, 2025). (d) RNAi‐mediated cleavage of a target gene results in downstream effects on the expression of other genes or direct off‐target effects. Such effects can be investigated using transcriptomics and proteomics to reveal changes in biological pathways. Exemplary data show how a dsRNA against the proteasome caused upregulation and downregulation of non‐target proteins (Cedden, Güney, Rostás, & Scholten, 2025). dsRNA, double‐stranded RNA; RISC, RNA‐induced silencing complex; RNAi, RNA interference; siRNA, small interfering RNA.

Most studies on RNAi‐based pest control focus on the lethal phenotype of dsRNA treatment, yet bioassays should ideally be complemented by molecular experiments to confirm induction of the RNAi pathway, since assays with certain insects can be difficult and background mortality may be misinterpreted as a dsRNA effect. The most common validation approach is measuring target gene expression via reverse transcription–quantitative polymerase chain reaction (RT‐qPCR), where reduced expression is expected in the corresponding dsRNA treatment group. However, while gene expression measurements can confirm RNAi pathway induction, they provide only limited insight into the underlying mechanism. Consequently, recent studies have increasingly adopted more sophisticated methods to both verify RNAi activity and gain a mechanistic understanding of RNAi‐based pest control (Figure 2 and Table 1).

**TABLE 1:** Experimental methods established for the study of RNAi‐based pest control.

Method	Purpose	Specialized kits	References
RISC‐bound small RNA‐seq	dsRNA processing and guide strand selection	TraPR Small RNA Isolation Kit (Lexogen)	(Cedden, Güney, Rostás, & Bucher, 2025; Grentzinger et al., 2020)
RNA degradomics	siRNA‐mediated transcript cleavage patterns	Not applicable	(Cedden, Güney, Rostás, & Scholten, 2025)
Transcriptomics and proteomics	Direct and indirect effects of dsRNA on cellular pathways	Not applicable	(Graser et al., 2025)
dsRNA visualization	dsRNA uptake and nonspecific degradation	Silencer™ siRNA Labelling Kit with Cy™3 dye (Thermo Fisher)	(Koo & Palli, 2024; Shi et al., 2026)

Abbreviations: dsRNA, double‐stranded RNA; RISC, RNA‐induced silencing complex; RNAi, RNA interference; siRNA, small interfering RNA.

### RISC‐bound small RNA‐seq investigates dsRNA processing and guides siRNA strand selection

Exogenous dsRNA is first recognized and processed by Dicer‐2 into ~21 nt siRNAs that are initially in duplex form. Next, the siRNA duplex is loaded onto RISC, which retains one of the siRNA strands, termed the guide strand, to find and cleave complementary transcripts. Hence, understanding how a given dsRNA is processed into siRNAs and which ones eventually become functional by being retained by RISC as the guide strand is of great importance for evaluating RNAi efficacy. Of particular interest is guide strand selection, namely, whether antisense or sense siRNA strands are enriched in RISC, since only antisense, not sense, siRNA strands are complementary to the intended target transcript.

Recently, a universal method for the isolation of RISC‐bound small RNAs has been developed, which provided opportunities for better understanding of dsRNA processing and guide strand selection (Grentzinger et al., 2020). The method depends on the isolation of RISC from tissues of interest, followed by the extraction and sequencing of associated small RNAs. Importantly, this method does not rely on immunoprecipitation but instead exploits conserved biochemical properties of Argonaute proteins, enabling broad applicability across laboratories and species. Typically, 5–10 mg of the biological material is sufficient, although successful small RNA isolation has been achieved from as little as 1–5 whole beetles, depending on size (Cedden, Güney, Rostás, & Bucher, 2025; Cedden, Güney, Rostás, & Scholten, 2025).

In RNAi‐based pest control, the RISC‐bound small RNA sequencing data can be mapped onto the delivered dsRNA sequence to investigate dsRNA‐derived siRNAs. The advantage of RISC‐bound small RNA‐seq compared to size‐selection based small RNA‐seq is that the former contains the actual functional siRNA, rather than nonfunctional siRNAs or target transcript degradation products that are indistinguishable from functional siRNAs via bioinformatic analysis.

The broad applicability of the method has already allowed investigation of RNAi based pest control in the coleopteran pest model *Tribolium castaneum*. In the study, this method was used to assess whether different dsRNAs optimized to contain more siRNA regions with favourable features, including weak 5′ and strong 3′ pairing of the antisense strand, minimal secondary structure, higher GC content between nucleotides 9–14 and an adenine at the 10th position, had a higher antisense‐to‐sense RISC‐bound siRNA ratio compared to dsRNA with less favourable features targeting the same target gene (Cedden, Güney, Rostás, & Bucher, 2025). Indeed, the analysis revealed ~ 20% difference in the antisense‐to‐sense ratio between optimized versus non‐optimized dsRNAs, and higher ratios were, in most cases, associated with higher lethal efficacy of the dsRNA treatment in the bioassays. The method can be further used to investigate tissue‐specific dsRNA processing patterns and whether various formulations, such as nanoparticles, can improve dsRNA processing in otherwise recalcitrant insect species, such as lepidopterans.

### RNA degradomics characterizes siRNA‐mediated transcript cleavages

A key step in the RNAi pathway that results in reduction of target gene transcript and protein abundance is the cleavage of the complementary transcript by dsRNA‐derived siRNAs. Although methods such as RT‐qPCR, transcriptomics and proteomics can confirm the downstream effects of transcript cleavage, they cannot investigate the actual cleavage events. To that end, recent studies have adapted RNA degradomics, also known as parallel analysis of RNA ends (PARE), a commonly used method for characterizing endogenous gene regulation in plants, to study the mode of action of insecticidal dsRNA in insect pests.

The method works by capturing transcript fragments that do not contain the 5′‐7‐methylguanosine cap present in intact mRNA and attaching an RNA adaptor at the 5′ cleavage sites. Poly‐A tail‐dependent library preparation and sequencing are performed to generate cleavage (or more generally degradation) frequency plots by mapping sequencing data onto transcript sequences. In parallel, the dsRNA‐derived siRNAs corresponding to the cleavage hotspots are analyzed to investigate siRNA–transcript cleavage dynamics. Currently, bioinformatics pipelines for plant miRNAs, which behave similarly to insect siRNAs, including CleaveLand and PAREsnip (Morgado & Johannes, 2019), allow this analysis in insect pests, although insect‐specific tools should be developed in future studies to improve accuracy.

Using RNA degradomics in combination with RISC‐bound small RNA sequencing, a recent study in the cabbage stem flea beetle, *Psylliodes chrysocephala*, showed that oral uptake of dsRNA against a proteasome subunit results in a key cleavage event between a uracil and guanine, which is likely responsible for the severe insecticidal effects of the treatment (Cedden, Güney, Rostás, & Scholten, 2025). RNA degradomics may be further used in future studies on diverse insect pests to improve the efficacy of dsRNAs in guiding cleavages and enhancing pest control efficacy. Specifically, RNA degradomics allows the identification of regions in target transcripts that are more susceptible to RNAi‐mediated cleavage, and this knowledge can be exploited for optimizing target sequences.

Another interesting possibility is to use RNA degradomics to investigate the cleavage or nonspecific degradation of dsRNA itself. This would require a slight adjustment to the standard protocol, since the capture method relies on poly‐A tails of mRNA transcripts. With appropriate adaptation, the method could provide previously inaccessible insights into dsRNA processing and degradation by nonspecific nucleases, which could shed light on poorly studied aspects of RNAi. For instance, RNA degradomics could reveal whether particular regions of the dsRNA are more susceptible to nonspecific cleavage by gut nucleases. These could be distinguished from canonical Dicer‐2‐mediated cleavages based on fragment length and whether the cleavage occurs on double‐ or single‐stranded RNA. Moreover, it is currently assumed that the siRNA pool directly results from dsRNA processing by Dicer‐2. However, additional dynamics may be at play, such as the differential half‐life of siRNAs, which could ultimately determine the mature RISC‐bound siRNA pool alongside the initial dsRNA processing by Dicer‐2. RNA degradomics targeting dsRNA, coupled with sequencing of RISC‐bound small RNAs, could help elucidate these effects.

### Transcriptomics and proteomics reveal genome‐wide effects of dsRNA

The siRNA‐guided transcript cleavage results in depletion of the target transcript and subsequently the corresponding protein level in the cell. Moreover, the dsRNA may have direct off‐target effects on unintended transcripts, or depletion of the target transcript may indirectly influence the expression of other transcripts and proteins through gene regulatory networks (Cedden & Bucher, 2025; MacNeil & Walhout, 2011). For these reasons, it is worthwhile to assess the genome‐wide effects of dsRNA treatment rather than solely validating target transcript depletion. To that end, transcriptomics and proteomics, commonly used in gene regulation research, have been integrated into RNAi‐based pest control studies.

A recent paper used both transcriptomics and proteomics to investigate the effects of the commercial dsRNA Ledprona against the proteasome subunit PSMB5 in *L. decemlineata* (Graser et al., 2025). In addition to confirming dsRNA‐mediated suppression of PSMB5 mRNA and PSMB5 proteins, the analyses identified compensatory upregulation of transcripts encoding other proteasome subunits and accumulation of ubiquitinated protein waste, indicating impairment of proteasome function. Similarly, another recent study in *P. chrysocephala* identified changes in protein levels following oral uptake of dsRNA targeting the proteasome subunit Prosβ1, including an increase in mitochondria‐ and cytoskeleton‐related proteins and a decrease in proteins associated with transcription and translation (Cedden, Güney, Rostás, & Scholten, 2025). These studies demonstrate that transcriptomics and proteomics can reveal important insights into the cellular effects of insecticidal dsRNA. Furthermore, identification of compensatory upregulation following RNAi induction suggests potential strategies to increase RNAi‐based pest control efficacy through co‐targeting of the primary target gene and compensatory genes.

Transcriptomics, in particular, has become increasingly accessible in insect research. Consequently, studies focusing on off‐target effects of RNAi‐based pest control can readily integrate transcriptomics to examine genome‐wide effects. For instance, one study used transcriptomics to assess the effects of insecticidal siRNAs in both target and non‐target insects (Ma et al., 2022). Interestingly, the study found that changes in gene expression did not fully correlate with in silico complementarity between siRNAs and transcripts, suggesting that experimental approaches are necessary to comprehensively characterize off‐target effects. Moreover, combining transcriptomics with RNA degradomics may help distinguish between direct off‐target effects, such as siRNA‐mediated transcript cleavage, and indirect effects mediated through gene regulatory networks in both target pest species and non‐target organisms.

In addition to sequence‐dependent effects, dsRNA itself can trigger sequence‐independent responses. For instance, dsRNA can act as a pathogen‐associated molecular pattern, activating innate immune pathways such as Toll, which can lead to the induction of immune genes independent of sequence (Feng et al., 2026; Liu et al., 2013). Exogenous dsRNA can also upregulate the expression of core RNAi genes, including Dicer‐2 and Argonaute‐2 (Kolliopoulou et al., 2015). Although transcriptomics and proteomics are well‐suited to characterize the full extent of these sequence‐independent effects of exogenous dsRNA, they remain underused for this purpose. Future studies could therefore provide important insights into these effects.

### dsRNA visualization

RNAi‐based pest control crucially depends on the uptake of dietary dsRNA into insect cells. The response to dietary dsRNA is highly variable among insect orders and even within orders (Shukla et al., 2016; Willow & Veromann, 2021). Coleopterans, especially leaf beetles (Chrysomelidae), are highly responsive to orally delivered dsRNA, making them ideal candidates for RNAi‐based pest control (Baum et al., 2007; Buer et al., 2025), while most lepidopterans display poor uptake and inefficient systemic spread of dsRNA, limiting the applicability of this approach in these taxa (Shukla et al., 2016; Terenius et al., 2011). Given that the core RNAi machinery is present in virtually all insects, the variable responses to dietary dsRNA are attributable to other factors, including rapid degradation of dsRNA by non‐specific nucleases in the gut lumen, inability of cells to import dsRNA and trapping of dsRNA in endosomes (Huvenne & Smagghe, 2010; Koo & Palli, 2025). To elucidate bottlenecks upstream of the core RNAi pathway that could explain the high variability in dsRNA responses, dsRNA visualization methods have been applied in insect pests.

To accurately visualize dsRNA, it needs to be labelled and observed using a fluorescence microscope. A particularly accessible dsRNA labelling approach is Cy3 (Cyanine3), an orange fluorescent dye (Naganuma et al., 2021). Long or short dsRNA can be Cy3‐labelled through co‐incubation and delivered to the insect of interest. Next, tissues of interest such as the gut are investigated, ideally using confocal microscopy. This method allows localization of dsRNA in extracellular compartments such as the gut lumen, cellular surfaces and subcellular compartments. This, in turn, can reveal whether dsRNA is efficiently taken up into cells and released into the cytoplasm. Because fluorescent nucleotides are typically incorporated at multiple positions along the dsRNA during synthesis, the signal is generally retained even after partial degradation of the dsRNA. However, fluorescent fragments or siRNAs derived from labelled dsRNA may confound interpretation. Therefore, dsRNA labelling‐based approaches should be complemented by other methods such as RISC‐bound small RNA sequencing to assess dsRNA processing.

Using Cy3‐labeled dsRNAs, a recent study in *L. decemlineata* cells showed that endocytosed dsRNA is routed through the endosome–Golgi retrograde pathway into the endoplasmic reticulum (Koo & Palli, 2024). The dsRNA‐binding protein StaufenC localizes to the endoplasmic reticulum and interacts with endoplasmic reticulum‐associated degradation (ERAD) proteins to release dsRNA into the cytosol, where recognition by Dicer‐2 can initiate the RNAi pathway. Knockdown of StaufenC trapped dsRNA in the endoplasmic reticulum and abolished siRNA production, whereas its overexpression enhanced RNAi in otherwise recalcitrant Sf9 cells derived from a lepidopteran.

In another recent study, the authors used dsRNA with fluorescein‐labelled UTP to investigate cellular uptake of extracellular dsRNA in different tissues of the migratory locust, *Locusta migratoria* (Shi et al., 2026). The investigation revealed that dsRNA uptake varies by tissue and involves clathrin‐mediated, caveolin‐mediated, macropinocytic and Sid‐like channel pathways, with clathrin‐mediated endocytosis emerging as the most broadly conserved mechanism across insect species.

These studies show that dsRNA visualization is an insightful approach for investigating how dsRNA is taken up and made available to the RNAi machinery and can help guide the development of strategies to enable RNAi‐based control of currently recalcitrant pest species.

## BIOINFORMATIC APPROACHES

Basic research and practical applications exploiting the RNAi pathway are based on the design of RNA sequences (e.g., siRNA and dsRNA) to effectively achieve target gene silencing in the organism of interest. For this reason, bioinformatic analysis of sequences used in RNAi applications has been a considerable focus from very early on. Development of specialized bioinformatics tools for RNAi applications has greatly benefited from general advances in the bioinformatics field, including the development of sequence alignment and RNA structure prediction tools (Langmead et al., 2009; Lorenz et al., 2011).

A comprehensive bioinformatic pipeline for RNAi‐based pest control should consider three main aspects: target gene identification, dsRNA efficacy and potential off‐target effects. Early RNAi tools were developed with a focus on siRNA therapeutics and basic research. The comprehensive ones, including DEQOR (Henschel et al., 2004) and E‐RNAi (Horn & Boutros, 2010), predicted efficacy and minimized off‐targets in humans or model organisms (Table 2). These tools have also been used to some degree in the pest control literature. For instance, DEQOR was used during the designs of dsRNA for the genome‐wide RNAi screen in *T. castaneum* (Ulrich et al., 2015). However, these tools have several limitations that render them suboptimal for use in RNAi‐based pest control. First, these tools are based on data in human cells. In addition, most, if not all, of these early web‐based tools have either become unavailable to researchers due to lack of maintenance or commercialization. Additionally, RNAi‐based pest control has special considerations that are not covered by these tools.

**TABLE 2:** Bioinformatics tools relevant for RNAi‐based pest control research.

Tool	Availability	Functions	Limitations
dsRIP web platform (Cedden, Güney, Rostás, & Bucher, 2025)	Web application: https://dsrip.uni‐goettingen.de	Insect‐data driven target gene prediction, insect‐data based dsRNA optimization, essential off‐target gene prediction, primer design	Off‐target predictions need to be experimentally tested, testing in non‐coleopterans is needed
dsRNAmax (Fletcher et al., 2025)	Local tool	Multi‐species targeting dsRNA, off‐target prediction	No efficacy prediction, off‐target gene function is not considered, usage requires bioinformatics expertise
dsRNAEngineer (Chen et al., 2025)	Web application: https://dsrna‐engineer.cn/	Multi‐species targeting dsRNA, off‐target prediction	No efficacy prediction, off‐target gene function is not considered
dsOMG (Lyu et al., 2025)	Web application: https://dsomg.sysu.edu.cn/	Off‐target prediction	Off‐target gene function is not considered, no considerations other than off‐target prediction
si‐Fi (Lück et al., 2019)	Local tool	Off‐target prediction, siRNA efficacy prediction	Validated only in plants, based on human data
iBeetle‐Base (Dönitz et al., 2015)	Web application: https://ibeetle‐base.uni‐goettingen.de/	Gene lethality data from a genome‐wide RNAi screen, contains database of tested dsRNA sequences	Only supports *Tribolium castaneum*, provides no functionality for novel dsRNA design
E‐RNAi (Horn & Boutros, 2010)	Web application: https://e‐rnai.dkfz.de/signaling/e‐rnai3	siRNA efficacy prediction, siRNA off‐target prediction, primer design	dsRNA design function is unavailable at least since 2022, limited species for off‐target prediction, off‐target gene function is not considered, based on human data
dsCheck (Naito et al., 2005)	Web application: https://dscheck.rnai.jp/	Off‐target prediction	Limited species for off‐target prediction, gene function is not considered, no considerations other than off‐target prediction
DEQOR (Henschel et al., 2004)	Proprietary tool (previously available as web application)	siRNA efficacy prediction, siRNA off‐target prediction	Publicly unavailable at least since 2022, based on human data

Abbreviations: dsRNA, double‐stranded RNA; RNAi, RNA interference; siRNA, small interfering RNA.

Recent developments highlight a trend toward accessible and user‐friendly RNAi‐based pest control‐focused platforms (Table 2). The dsRIP web platform (https://dsrip.uni-goettingen.de/) and dsRNAEngineer (https://dsrna-engineer.cn/) are prominent examples of this trend (Cedden, Güney, Rostás, & Bucher, 2025; Chen et al., 2025). These developments indicate growing interest in standardized computational pipelines in the field and suggest that knowledge of RNAi in pests has reached a threshold that makes computational implementations feasible.

### Effective target gene selection

Inducing the RNAi pathway via the delivery of dsRNA results in the depletion of complementary mRNAs. In principle, the mRNA of any gene among more than 14,000 genes possessed by a typical insect genome may be targeted via this strategy (Cabañas et al., 2025; Richards et al., 2008). However, most eukaryotic genes are either redundant, or their functions are not essential. As the principle of RNAi‐based pest control is to induce severe insecticidal effects, it is clear that only particular target genes are suitable for RNAi‐based pest control. Work in *D. melanogaster* has demonstrated that roughly one‐third of its genes (~5000 genes) are essential, meaning their disruption leads to lethality at some point during its life cycle under laboratory conditions (Nüsslein‐Volhard, 1994). Although this large pool of essential genes makes it relatively easy to identify potential targets, the real difficulty is selecting those that produce strong phenotypes after minimal dsRNA delivery, a requirement for practical field applications. To determine an effective target gene for RNAi‐based pest control, bioinformatics approaches are used to transfer essential genes from a reference organism (e.g., a model organism) through orthology inference.

iBeetle‐Base offers an invaluable resource for the identification of effective target genes as it includes lethality data from an RNAi screen where dsRNA injection against virtually all protein‐coding genes was tested in *T* (Buer et al., 2025; Dönitz et al., 2015). This screen showed that targeting conserved core cellular processes, such as proteasome and gene expression pathways, is more effective than targeting typical chemical insecticide targets, such as the nervous system. Using this resource, effective target genes can be identified efficiently in insects, particularly in coleopterans, via orthologous pest gene identification (Buer et al., 2025; Canuto et al., 2025; Cedden, Güney, Rostás, & Debaisieux, 2025; Mehlhorn et al., 2021).

To robustly determine orthologs, rigorous orthology inference methods should be used rather than relying solely on BLAST searches of nucleic acid sequences. Amino acid sequences typically provide more reliable functional inference than nucleotide sequences, and reciprocal searches are important for accurate results (Emms & Kelly, 2019). Applying robust orthology frameworks expands the pool of candidate essential genes and supports more effective target selection. Recent robust orthology inference pipelines applicable to many insect pests at scale include OrthoFinder and FastOMA (Emms & Kelly, 2019; Majidian et al., 2025). The prerequisite for orthology inference is a transcriptome or genome‐extracted coding sequences (CDS), which can be converted to amino acid sequences as input.

The dsRIP web platform offers unprecedented ease in effective target gene identification in pests by combining curated genome‐wide RNAi screen data with automated orthology inference in a user‐friendly manner (Cedden, Güney, Rostás, & Bucher, 2025). First, dsRIP already includes ~100 important pest species for which orthology inference has been conducted, and users can select effective target gene orthologs from *T. castaneum* RNAi screen data through a graphical interface on the website. Optional filtering parameters allow users to filter candidate targets based on successful transfers to other pest species in previous studies. Moreover, users can analyse any pest species of interest either by automatically retrieving genomic resources from NCBI or by uploading a custom FASTA file containing transcripts or CDS. The recently developed tool dsRNAEngineer also provides target gene selection functionality, but with a distinct objective (Chen et al., 2025). Its focus is the identification of target genes with high sequence similarity within and across species for designing co‐targeting dsRNA.

### dsRNA sequence optimization

Following the identification of a target gene, the dsRNA sequence must be designed. Typically, it is made fully complementary to a specific region in the mRNA. Based on this design, the sense strand (matching the selected mRNA region) and the antisense strand (reverse complementary to the selected mRNA region) are synthesized and annealed to form the dsRNA.

Until recently, the only well‐established rule during the design of dsRNAs for RNAi‐based pest control concerned the length. dsRNAs shorter than 60 base pairs are not effectively taken up by insect cells, which would prevent the induction of the RNAi pathway. dsRNA uptake improves with length up to about 200 bp (Bolognesi et al., 2012; He et al., 2020; Koo & Palli, 2025; Miller et al., 2012). For this reason, dsRNA designs are usually made longer than 200 bp. Length is a straightforward parameter that can be easily incorporated into dsRNA design. The subsequent question is how to select the actual dsRNA target region within the mRNA. A typical mRNA is around 2000 bp in length, while dsRNAs are generally between 200 and 500 bp, leaving a notable degree of freedom in target region selection.

A recent study in *T. castaneum* investigated how siRNA features influence the efficacy of designed dsRNAs (Cedden, Güney, Rostás, & Bucher, 2025). The results showed that high efficacy correlated with thermodynamic asymmetry, that is, weak 5′ and strong 3′ pairing of the antisense strand, minimal secondary structure, higher GC content between nucleotides 9 and 14, adenine at the 10th position and targeting of the open reading frame of the mRNA. Enriching these siRNA features during dsRNA design improved the antisense‐to‐sense siRNA ratio in the RISC and enhanced the insecticidal efficacy of dsRNA targeting essential genes.

Based on these findings, an algorithm was developed to optimize dsRNA sequences. This algorithm is integrated as the core functionality of the dsRIP web platform. Users can select recommended target genes or input custom gene sequences to design optimized dsRNAs within a desired length range. The advantage of dsRIP's dsRNA optimization algorithm compared to earlier tools is that it uses insect pest‐optimized rather than human cell‐based siRNA parameters for dsRNA optimization, and it is currently publicly available (Table 2).

In contrast, dsRNAmax and dsRNAEngineer do not attempt to predict intrinsic siRNA efficacy (Chen et al., 2025; Fletcher et al., 2025). Instead, they enable the rational design of chimeric dsRNAs intended for multi‐gene and multi‐species targeting. For instance, dsRNAmax implements a k‐mer‐driven assembly strategy in which constructs are optimized to maximize the number and distribution of siRNA‐sized subsequences shared across related targets. Rather than selecting a single contiguous region from one transcript, the algorithm generates a chimeric template by iteratively extending overlapping k‐mers (21 nt by default), prioritizing those most frequently represented among the input target sequences. The final dsRNA is then selected to maximize the median abundance of matching k‐mers across all targets. This process produces a mosaic‐like construct that distributes silencing potential across sequence diversity, allowing a single dsRNA molecule to target multiple homologous RNAs. The design logic therefore emphasizes breadth of coverage and tolerance to natural variation rather than dependence on any single locus. These tools are expected to facilitate the development of RNAi‐based products targeting multiple pest species simultaneously, a strategy that has not reached commercialization.

### Off‐target prediction for safer dsRNA design

A special case of off‐target prediction arises in the context of pest control: the identification of off‐targets, not in the target pest itself, but non‐target organisms (Chen et al., 2021; Devisetty et al., 2025; Mogren & Lundgren, 2017). A non‐target organism refers to a species that shares the same environment as the target pest (e.g., a crop field) and would therefore also be exposed to dsRNA‐based applications. Notable non‐target organisms include honey bees in flowering crops and predatory ladybugs (i.e., most coccinellids). If the applied dsRNA contains regions that significantly overlap with genes in these non‐target organisms, there is a risk of detrimental effects. Hence, predicting potential off‐targets and designing dsRNA sequences with the lowest possible risk are of great importance.

In practice, high complementarity is defined by the number of mismatches between the siRNA and off‐target transcripts relative to a perfectly matched sequence based on canonical base pairing (i.e., A–U and G–C). Typically, sequences with perfect complementarity or up to two mismatches are considered to have the potential to induce off‐target effects. Given the typical siRNA length of 21 nt, the presence of at least 19 complementary nucleotides to an off‐target transcript is sufficient to flag the siRNA as a potential source of off‐target activity. This rationale is based on the ability of the RISC to tolerate a small number of nucleotide mismatches when recognizing transcripts complementary to the guide strand (Chen et al., 2021; Kulkarni et al., 2006).

Most recent tools, including dsRIP, dsRNAMAX, dsRNAEngineer and dsOMG (Table 2), offer off‐target prediction functionality aimed at improving the environmental safety of dsRNA designs. In general, these tools converge on a similar off‐target prediction pipeline, where the input sequence is first processed into all possible siRNAs, which are then searched against the transcriptomes of selected non‐target organisms. Sequence alignment tools such as BLAST and Bowtie are commonly used to predict potential off‐targets in the non‐target organisms under consideration and to minimize them during dsRNA design (Devisetty et al., 2025). Through this process, siRNAs with high complementarity to off‐target transcripts are identified, and the design algorithm avoids incorporating such siRNAs into the final dsRNA construct.

Some tools also introduce special considerations for off‐target prediction during dsRNA design. For instance, dsRNAMAX and dsRNAEngineer can simultaneously consider multi‐targeting of intended pest species while still minimizing off‐targets in non‐target organisms (Chen et al., 2025; Fletcher et al., 2025). Similarly, dsRIP considers both efficacy and potential off‐target effects and allows priority input from the user (Cedden, Güney, Rostás, & Bucher, 2025). Moreover, dsRIP provides an additional functionality for predicting off‐target genes that are likely to be essential, based on genome‐wide RNAi screening data. In its prioritization scheme, dsRIP places greater emphasis on avoiding off‐targets involving essential genes in the selected non‐target organisms, as these pose a higher risk of detrimental effects. Finally, both dsRIP and dsRNAEngineer support the upload of custom sequences for off‐target prediction, which is particularly advantageous when working with poorly characterized pest species.

Off‐target prediction pipelines may be improved in biological relevance by accounting for the positional distribution of mismatches and their influence on RISC activity, as well as by considering non‐canonical base pairing (e.g., G–U wobble pairs), which may partially compensate for mismatches.

## OUTLOOK

RNAi‐based pest control is transitioning from proof‐of‐concept studies toward practical applications in the field. The integration of molecular methodologies, such as RISC‐bound small RNA sequencing and RNA degradomics, is likely to redefine how efficacy and safety are evaluated, shifting emphasis from phenotype‐centric bioassays to mechanistic interpretation. As these approaches become more routine, discrepancies between observed lethality and underlying RNAi activity can be resolved with greater confidence, improving both experimental reproducibility and risk assessment.

Future progress will depend on resolving biological constraints that currently limit RNAi performance in many insect taxa. Systematic comparisons across responsive and recalcitrant species, particularly when combined with dsRNA visualization and degradomics, may reveal bottlenecks governing uptake, intracellular trafficking and nuclease activity. Such knowledge will inform rational formulation strategies, including carrier systems and chemical modifications designed to enhance dsRNA stability and cytosolic delivery. Mechanistic clarity at this level is essential for extending RNAi applications beyond highly responsive groups such as coleopterans.

On the computational side, emerging insect‐focused design platforms signal a maturation of the field toward standardized, data‐driven pipelines. Continued refinement of these tools, supported by expanding genomic resources and experimentally validated datasets, should improve target selection, efficacy prediction and safety evaluation. Importantly, predictive algorithms will require iterative feedback from molecular and organismal experiments to avoid overfitting to limited model systems. The reciprocal development of experimental and bioinformatic frameworks will therefore remain central to advancing predictability in RNAi applications, strengthening regulatory confidence and public acceptance.

## AUTHOR CONTRIBUTIONS

**Doga Cedden:** Conceptualization; writing – original draft; writing – review and editing.

## CONFLICT OF INTEREST STATEMENT

The author declares no conflicts of interest.

## Data Availability

Data sharing not applicable to this article as no datasets were generated or analysed during the current study.
